# Protein kinase B/AKT phosphorylates hypoxia-inducible factor-3α1 in response to insulin, promoting cell growth and migration

**DOI:** 10.3389/fcell.2023.1250000

**Published:** 2023-11-09

**Authors:** Tran Vinh Hong Nguyen, Ulrich Bergmann, Thomas Kietzmann, Daniela Mennerich

**Affiliations:** Faculty of Biochemistry and Molecular Medicine, and Biocenter Oulu, University of Oulu, Oulu, Finland

**Keywords:** HIF-3α, insulin, PKB/AKT, hypoxia, phosphorylation, cell migration

## Abstract

Hypoxia-inducible factors (HIFs) are best known for their roles in the adaptation to low oxygen environments. Besides hypoxia, HIF-1/2 α-subunits are also regulated by various non-hypoxic stimuli including insulin which can act via the PI3K/protein kinase B (PKB) signaling pathway. However, with respect to insulin little is known about HIF-3α. We aimed to investigate this relationship and found that insulin stimulates HIF-3α expression under both normal and low oxygen conditions. Blocking PKB activity reversed the effects of insulin, indicating that HIF-3α is a direct target of PKB. We identified serine 524, located in the oxygen-dependent degradation domain of HIF-3α, as a phosphorylation site of PKB. Mutating serine 524 impaired binding of PKB to HIF-3α and its ubiquitination, suggesting that PKB regulates HIF-3α stability through phosphorylation, thereby affecting important cellular processes such as cell viability and cell adhesion. Importantly, we discovered that this phosphorylation site also influenced insulin-dependent cell migration. These findings shed light on a novel mechanism by which insulin affects PKB-dependent HIF-3α expression and activity, with potential implications in metabolic diseases and cancer.

## 1 Introduction

Transcriptional gene regulation in response to hypoxia is mediated mainly by proteins from the hypoxia-inducible factor (HIF) family. Thereby various cellular functions such as glucose metabolism, hematopoiesis, angiogenesis, cell survival and death are adapted to the changed oxygen availability (Kaelin and Ratcliffe, 2008; Semenza, 2010).

HIFs are heterodimeric transcription factors that consist of an α-subunit that is destabilized in the presence of oxygen and a constitutively expressed β-subunit [HIF-1β, also known as aryl hydrocarbon receptor nuclear translocator (ARNT)] (Wang et al., 1995; Wang and Semenza, 1995). Three HIFα subunits are known to date. In this context, HIF-1α and HIF-2α have overlapping but non redundant roles with respect to hypoxia-induction kinetics as well as tissue and target gene specificity (Holmquist-Mengelbier et al., 2006; Keith et al., 2011; Koh et al., 2011).

Unlike the other two subunits, HIF-3α is different. Indeed, the transcription of the *HIF3A* gene seems to be more complex compared to the *HIF1A* and *EPAS1* (encoding HIF-2α) genes. Several alternative transcription sites can be used, and the transcripts are largely subjected to alternative splicing. As a result, mice mainly express three variants, namely, HIF-3α, inhibitory PAS domain (IPAS), and neonatal embryonic PAS (NEPAS) (Gu et al., 1998; Makino et al., 2001; Yamashita et al., 2008) whereas 10 different HIF-3α variants have been detected in humans so far (Maynard et al., 2003; Pasanen et al., 2010). Among those, HIF-3α1 encodes the full-length protein which is shorter than HIF-1α and HIF-2α and consists of 667 amino-acids (Gu et al., 1998; Hara et al., 2001).

All full-length HIFα subunits belong to the basic helix-loop-helix (bHLH) PER-ARNT-SIM (PAS) protein family and contain an oxygen-dependent degradation domain (ODDD). The latter is of particular importance for the O_2_-dependent regulation of protein stability. In the presence of O_2_, this domain is hydroxylated by HIF proline hydroxylases. The hydroxylated prolines then serve as recognition tags for an E3-ubiquitin ligase complex containing the von-Hippel-Lindau protein (pVHL). The resulting polyubiquitylated HIFα subunits are then directed to proteasomal degradation (Kaelin and Ratcliffe, 2008; Kaelin, 2017). In addition to the ODDD, HIF-1α and HIF-2α contain two transactivation domains, the N-terminal domain (N-TAD) and the C-terminal domain (C-TAD) (Ivan et al., 2001; Jaakkola et al., 2001; Lando et al., 2002b). The transactivity of the C-TAD is also subject of O_2_-dependent hydroxylation. In this process, the Factor-Inhibiting HIF (FIH) hydroxylates a certain asparagine residue, which thereby can no longer bind the CBP/p300 co-activators (Lando et al., 2002a). In contrast, HIF-3α1 lacks the C-TAD but possesses a C-terminal leucine zipper domain (Gu et al., 1998; Hara et al., 2001; Kietzmann et al., 2001).

In addition to polyubiquitination which is quintessential for preventing HIFα accumulation in normoxia, other post-translational modifications such as phosphorylation, acetylation, methylation or sumoylation constitute another essential regulatory mechanism for the HIF-1α-and HIF-2α subunits (Dimova et al., 2009; Kietzmann et al., 2016; Albanese et al., 2021).

Insulin is a hormone that regulates various cellular processes, including glucose metabolism, cell proliferation and cell migration. The majority of insulin actions are mediated by posttranslational changes, mainly phosphorylation, of key regulators involved. Insulin has been shown to regulate HIF-1α (Treins et al., 2002; Flügel et al., 2007) and HIF-2α (Wang et al., 2019) both of which play significant roles in cell metabolism, growth and differentiation [for review, see (Schito and Semenza, 2016; Taylor and Scholz, 2022)]. While the landscape of post-translational modification of HIF-1α and HIF-2α is steadily expanding, current knowledge with respect to HIF-3α is limited. To date, it is known that 2-deoxy-D-glucose and insulin can increase HIF-3α expression in rat kidney, cerebral cortex (Heidbreder et al., 2007) and primary hepatocytes (Kietzmann et al., 2003). Since insulin is a major activator of the phosphoinositide 3-kinase-protein kinase B/AKT (PI3K-PKB/AKT) pathway, this may imply that insulin exerts its effect on HIF-3α via the PI3K-PKB/AKT axis, similar to FOXO transcription factors. HIF-3α could potentially be a direct PKB/AKT target during this process, which in turn modulates cell growth and migration.

Here we show that insulin stimulates the accumulation of HIF-3α1 under both normoxia and mild hypoxia in a PKB-dependent manner, as this induction could be antagonized by using a specific PKB inhibitor. Furthermore, our data reveal that PKB/AKT interacts with full-length HIF-3α1 and phosphorylates it at serine 524 in the oxygen-dependent degradation domain (ODDD). This phosphorylation has functional consequences as it affects colony formation, adhesion, and migration of cells. Overall, our data provide evidence that HIF-3α1 plays a role in cell growth and suggest that PKB/AKT directly regulates HIF-3α1 activity and function.

## 2 Materials and methods

### 2.1 Materials

All biochemicals and enzymes were of analytical grade and were obtained from commercial suppliers. Insulin (dissolved in 0.9% NaCl), Lithium chloride (dissolved in H_2_O), Wortmannin and Rapamycin were purchased from Sigma Aldrich. BML-257 and MG132 were purchased from Cayman chemicals. Unless otherwise stated, all substances were dissolved in DMSO.

### 2.2 Cell culture

HEK 293 and HepG2 cells were cultured under normoxia (16% O_2_, 79% N_2_, and 5% CO_2_ [v/v]) in MEM supplemented with 10% FBS. MCF-7 cells were cultured under normoxia (16% O_2_, 79% N_2_, and 5% CO_2_ [v/v]) in DMEM containing 10% FBS.

All cell lines were tested *mycoplasma* negative by using the MycoAlert Detection Kit (Lonza). In all experiments the number of cell passages used was below 10. For protein extraction, cells were seeded into 60 mm dishes and, when necessary, transfections were performed the next day for 12 h. Prior to treatment, cells were starved in serum-free media for 16 h, then treated either alone or in combination with 100 nM insulin, 20 nM wortmannin, 12.5 µM BML-257, 10 nM rapamycin or 500 nM LiCl and further cultured for 4 h under normoxia or mild hypoxia (5% O_2_, 90% N_2_, and 5% CO_2_ [v/v]). In all experiments control cells were treated with DMSO.

### 2.3 Plasmid constructs

The construct for full-length HIF-3α1 was amplified by polymerase chain reaction (PCR) from HepG2 cDNA using the following primer set: human HIF-3α1-F 5′- cac​cat​gga​ctg​gca​aga​c-3′ and human HIF-3α1-R 5′- gtc​agc​ctg​ggc​tga​gcc​tg-3′. The resulting PCR product was directly cloned into the pcDNA3.1D/V5-His-TOPO vector according to manufacturer’s protocol. The pcDNA6/Myc-His-Gal4-HIF-3α-TAD was generated by inserting a HindIII-BamHI Gal4DBD fragment (1–147) of the pSG424 (Sadowski and Ptashne, 1989) into the pcDNA6/Myc-His vector to create a pcDNA6/Myc-His-Gal4 construct. The HIF-3α-TAD (451–528) was amplified by PCR from HepG2 cDNA using the following primer set: TAD-HIF-3α-F (5′-CTG GGG ATC CAC AGA CTC TCC ACT GCC CGG-3′) and TAD-HIF-3α-R (5′-CCT GCT CGA GTC GAC TTG TCG TCG TCG TCC TTG TAG TCC GGC AAC AGG CCAT GGA AGC T-3′) and ligated into the BamHI and SalI sites of pcDNA6/Myc-His-Gal4 allowing generation of a Gal4 fusion protein. The construct for pG5E1B-Luc was described previously (Krüger et al., 1997). The vector expressing HA-tagged full-length PKB has been already described (Franke et al., 1995). The constructs for full-length HIF-3α1 and Gal4-HIF-3α-TAD with mutation in serine 524 were generated using the QuickChange mutagenesis kit (Promega). All constructs were verified by sequencing in both directions.

### 2.4 Cell transfection and luciferase assay

4 × 10^5^ cells per 60 mm dish were transfected as previously described (Roth et al., 2004). To investigate HIF-3α transactivity, cells were cotransfected with 2 µg of reporter construct pG5-E1B-Luc and 500 ng of the Gal4-HIF-3α-TAD or respective mutant construct. After 24 h, the medium was changed, cells were treated with insulin (100 nM) and further cultured in serum-free medium for 8 h under normoxic conditions. The detection of luciferase activity was performed with the Dual- Luciferase™ Reporter Gene Assay Kit (Berthold, Pforzheim, Germany).

### 2.5 Western blot analysis, protein half-life studies, immunoprecipitation, and ubiquitylation assays

Western blot analysis was carried out as described previously (Kietzmann et al., 1999). In brief, lysates from MCF-7, HEK 293 and HepG2 cells were collected, and 50–100 µg of protein was loaded onto a 7.5% or 10% sodium dodecyl sulfate (SDS)- polyacrylamide gel. After electrophoresis and electroblotting onto a nitrocellulose membrane, proteins were detected with monoclonal antibodies against V5 tag (#R960-25; 1:5,000; Invitrogen), hemagglutinin (HA-Tag #sc-7392; 1:500; Santa Cruz), phospho-GSK3β (Ser9) (#9323; 1:1,000; Cell Signaling), E-cadherin (#610181; 1:1,000; BD Biosciences), α-smooth muscle (#A2547; 1:1,000; Sigma Aldrich), ubiquitin (#646304; BioLegend; 1:1,000) or α-Tubulin (#T5168; 1:10,000; Sigma-Aldrich). Polyclonal antibodies were against phospho-AKT (Ser473) (#9271; 1:1,000; Cell Signaling), phospho-AKT (Thr308) (#9275; 1:1,000; Cell Signaling), total AKT (#9272; 1:1,000; Cell Signaling) or phospho-p70S6 Kinase (Thr421/Ser424) (#9204; 1:1,000; Cell Signaling). The secondary antibody was either an anti-mouse (#1706516) or an anti-rabbit immunoglobulin G conjugated (#1706515) to horseradish peroxidase (1:5,000; Bio-Rad). The ECL system (Amersham) was used for detection.

For half-life studies, HEK 293 cells were transfected together with expression vectors encoding full-length V5-tagged HIF-3α1 WT or mutant HIF-3α1 S524A. 48 h post transfection, cells were treated with protein synthesis inhibitor cycloheximide (CHX, 20 μg/mL, (Sigma-Aldrich)). Afterwards, cells were harvested at the indicated time points and protein levels were measured by immunoblot analysis. For immunoprecipitation and ubiquitylation assays, HEK 293 cells were treated with proteasome inhibitor MG 132 (50 μM; Calbiochem). Four hours later, cells were scraped in lysis buffer (50 mM Tris/HCL, pH 7.5, 150 mM NaCl, 1% Triton X-100, 2 mM EDTA, 2 mM EGTA, 1 mM PMSF and complete protease inhibitor cocktail tablet (Roche)). After scraping, lysates were incubated with continuous shaking at 4°C for 20 min and centrifuged at 12.000 *g* at 4°C for 15 min. To recover anti-V5 and anti-HA immunoprecipitates, 150 µg of protein was incubated with 2 µg antibody for 1 h at 4°C before protein G Sepharose beads (30 µL per reaction mixture; #GE17-0,618-01; GE Healthcare) were added for 12 h. Thereafter, the beads were washed five times with lysis buffer and recovered, pellets were dissolved in 2 X Laemmli buffer, loaded onto a 7.5 % SDS gel, blotted and detected with antibodies against ubiquitin and V5- or HA-epitope.

### 2.6 Cell viability assay

Cell viability was assessed by a MTT [3-(4,5-dimethylthiazol-2-yl)-2,5-diphenyl tetrazolium bromide] reduction assay. MCF-7 or HEK 293 cells were transfected with expression vectors for full-length wild-type HIF-3α1 or HIF-3α1 S524A mutant, seeded in 24-well plates (5 × 10^4^ cells per well) and cultured under normoxia (16% O_2_). After 24 h cells were treated with insulin (100 nM) and further cultured in serum-free medium for 8 h under normoxic conditions (16% O_2_). Then 500 µL of MTT (4 mg/mL) was added to the wells and incubated at 37°C for 2 h. Formation of MTT–formazane was measured with a Tecan plate reader at a wavelength of 570 nm.

### 2.7 Colony formation assay and adhesion assay

For colony formation, MCF-7 or HEK 293 cells were transiently transfected with plasmids for full-length V5-tagged HIF-3α1 WT or mutant HIF-3α1 S524A. After transfection, cells were further cultured for 20 h under normoxia (16% O_2_) and then seeded into 6-well plates at a density of 2,000 cells/well. After 7 days, cells were fixed with 4% PFA and stained with crystal violet (Applichem). Plates were either scanned or images of the colonies were taken using the Leica MZFLIII microscope at original magnification ×40 and analyzed using the ImageJ software.

For adhesion assay, MCF-7 cells were transiently transfected with plasmids for full-length V5-tagged HIF-3α1 WT or mutant HIF-3α1 S524A. 24 h post transfection, cells were seeded into 6-well plates and grown under normoxia (16% O_2_) until they reached confluency (2–3 days). The 6-well plates were then transferred to an orbital shaker and rotated at 250 rpm for 8 h at 37°C. Control plates remained in the incubator. After rotation, the medium was aspirated from the cells followed by washing with 1x PBS and fixation with 4% PFA for 20 min at room temperature. The cells were briefly washed with 1x PBS and stained with crystal violet solution. Subsequently, the staining solution was aspirated, and the wells were rinsed with water to remove excess stain and air dried. Plates were scanned and analyzed using the ImageJ software for quantification.

### 2.8 Wound healing assay

For scratch wound assay, MCF-7 cells were transiently transfected with plasmids for full-length V5-tagged HIF-3α1 WT or mutant HIF-3α1 S524A. 24 h post transfection, cells were seeded onto 96-well Essen ImageLock Plates (Essen Bioscience) (3 × 10^3^ cells per well) in DMEM with 10% FBS. The following day the confluent cell monolayer was wounded with the 96 PTFE pin Wound Maker (Essen Bioscience), cells were washed with 1x PBS and further cultured in DMEM supplemented with 0.1% FBS and insulin (100 nM). The live wound closure was phase contrast-imaged for 72 h in 4 h intervals and the confluence analysis was performed using the IncuCyte software.

### 2.9 Purification of the GST-TAD-N fusion proteins

*Escherichia coli* BL21 (DE3) cells transformed with pGEX-GST-HIF-3α-TAD or pGEX-5X1 were grown at 37°C in Luria-Bertani media containing 100 µg of ampicillin/mL and induced with 0.1 mM isopropyl-β-D-thiogalactoside (IPTG) for 4 h. Cells were then harvested, and glutathione S-transferase (GST)-HIF-3α-TAD and GST proteins were prepared essentially as described previously (Liu et al., 2004). The integrity and yield of purified GST proteins were assessed by SDS-polyacrylamide gel electrophoresis followed by Coomassie blue staining.

### 2.10 *In vitro* HIF phosphorylation assay

The purified wild-type or mutant GST-HIF-3α-TAD-N fusion proteins (20 µg) were incubated in kinase buffer [0.2 M MOPS (morpholinepropanesulfonic acid; pH 7.4), 0.5 M EDTA, 0.1 M Mg(CH_3_COO)_2_] in the presence of active AKT1 (50 mU) (#SRP0353; Sigma Aldrich) and 1 μCi/μL [γ-^33^P]ATP (#NEG302H; PerkinElmer). After 30 min of incubation at 30°C, samples were loaded onto a 10% SDS gel, and after electrophoresis and blotting onto a polyvinylidene difluoride membrane, phosphorylated proteins were visualized by phosphorimaging.

### 2.11 RNA preparation, reverse transcription and quantitative real-time PCR

Isolation of total RNA was performed using RNeasy Mini Kit (Qiagen, Hilden, Germany) following the manufacturer’s instructions. Total RNA concentration and purity were measured on a NanoDrop spectrophotometer. One μg of total RNA was used for cDNA synthesis using iScript cDNA Synthesis Kit (Bio-Rad, München, Germany).

Quantitative real-time PCR was performed in an Applied Biosystems 7,500 Real-Time PCR System (Life Technologies, Helsinki, Finland) by using an Applied Biosystem Power SYBR^®^ green PCR master mix (Life Technologies). The following primer sets were used: human HIF-3α-F (5′-GTC​GGA​GAG​TAT​CGT​CTG​TGT​C-3′), human HIF-3α-R (5′-TCT​GCG​AGA​GTG​TTG​CTC​CGT​T-3′), human β-actin-F (5′-GTT​GTC​GAC​GAC​GAG​CG-3′) and human-β-actin-R (5′-GCA​CAG​AGC​CTC​GCC​TT-3′). The experiments for each data point were carried out in triplicate. The relative quantification of gene expression was determined using the ΔΔCt method (Livak and Schmittgen, 2001).

### 2.12 Mass spectrometry

Co-immunoprecipitation and Mass spectrometry were conducted as follows. HEK 293 cells were transfected with an expression vector encoding full-length V5-tagged HIF-3α1 WT and full-length HA-tagged PKB and incubated for 24 h. Cells were collected by scraping and lysed in lysis buffer containing 50 mM Tris/HCl (pH 7.5), 150 mM NaCl, 10 mM Na_4_P_2_O_7_, 1% Triton X-100, 2 mM EDTA, 2 mM EGTA, and complete protease inhibitor cocktail tablet (Roche). The lysate was incubated with continuous shaking at 4°C for 20 min and then centrifuged at 12,000 g for 15 min at 4°C. To recover anti-V5, 150 µg of protein was incubated with 2 µg of antibody for 1 h at 4°C, followed by the addition of protein G Sepharose beads (30 µL per reaction mixture; #GE17-0,618-01; GE Healthcare) for 12 h. The beads were washed five times with lysis buffer, and the protein was recovered. The pellets were dissolved in 2X Laemmli buffer and loaded onto a 10% SDS gel. Subsequently, the gel was washed briefly with ddH_2_O and stained with Coomassie Blue for 1 h at room temperature. After overnight destaining, the gel piece containing a ∼72 kDa polypeptide was excised. The gel piece was completely destained with 40 mM ammonium bicarbonate in 40% acetonitrile, treated with 20 mM DTT for 30 min at room temperature, and alkylated by adding 45 mM iodoacetamide (IAA) for 30 min at room temperature. The gel piece was washed once with the same solvent and twice with ddH_2_O. Trypsin (Sigma proteomics grade, 5 ng/μL in 40 mM ammonium bicarbonate/9% acetonitrile) was added (20 µL) and incubated overnight at room temperature.

For data acquisition, a Nano LC 1000 system (Thermo Scientific, United States) coupled with a Lumos Fusion Orbitrap mass spectrometer (Thermo Scientific, United States) was used. Samples were trapped onto a Symmetry C18 100Å 5 µm 0.180 × 20 mm precolumn and eluted over nanoEaseBEH C18 130Å 1.7 µm 0.075 × 150 mm analytical column. The LC conditions included a flow rate of 0.3 μL/min and a gradient from 97% solvent A (0.1% formic acid in water) to 35% solvent B (0.1% formic acid in acetonitrile) over 90 min. Mass spectrometry data were acquired in data-dependent acquisition (DDA) mode with survey scans at a resolution setting of 120.000 over a mass range of 375–1,500. The automatic gain control (AGC) target was set to 4e5, and the maximum fill time was under 100 ms. MS/MS interrogation was triggered for ions with multiple charges and intensity above 2.5e4 in the Orbitrap, while ions between 1e3 and 2.5e4 were analyzed in the ion trap. AGC for Orbitrap analysis was set to 5e4 with a maximum fill time of 200 ms and a resolution of 15,000. The ion trap was filled for a maximum of 300 ms with AGC set to 10.000. HCD fragmentation with 30% collision energy was used for MS/MS.

### 2.13 Statistical Analysis

Densitometry data were plotted as fold induction of relative density units, with the zero-value absorbance in each figure set arbitrarily to 100%. Statistical comparisons of absorbance differences were performed by the Mann-Whitney test (Statview 4.5, Abacus Concepts, Berkeley, CA), and *p* values *p* ≤ 0.05 were considered significant. Luc values presented are means +SEM. Results were compared by ANOVA for repeated Luc measurements followed by the Newman-Keuls test. A probability level *p* ≤ 0.05 was accepted as significant.

## 3 Results

### 3.1 Induction of HIF-3α1 protein expression by insulin via protein kinase B (PKB)

**FIGURE 1:**
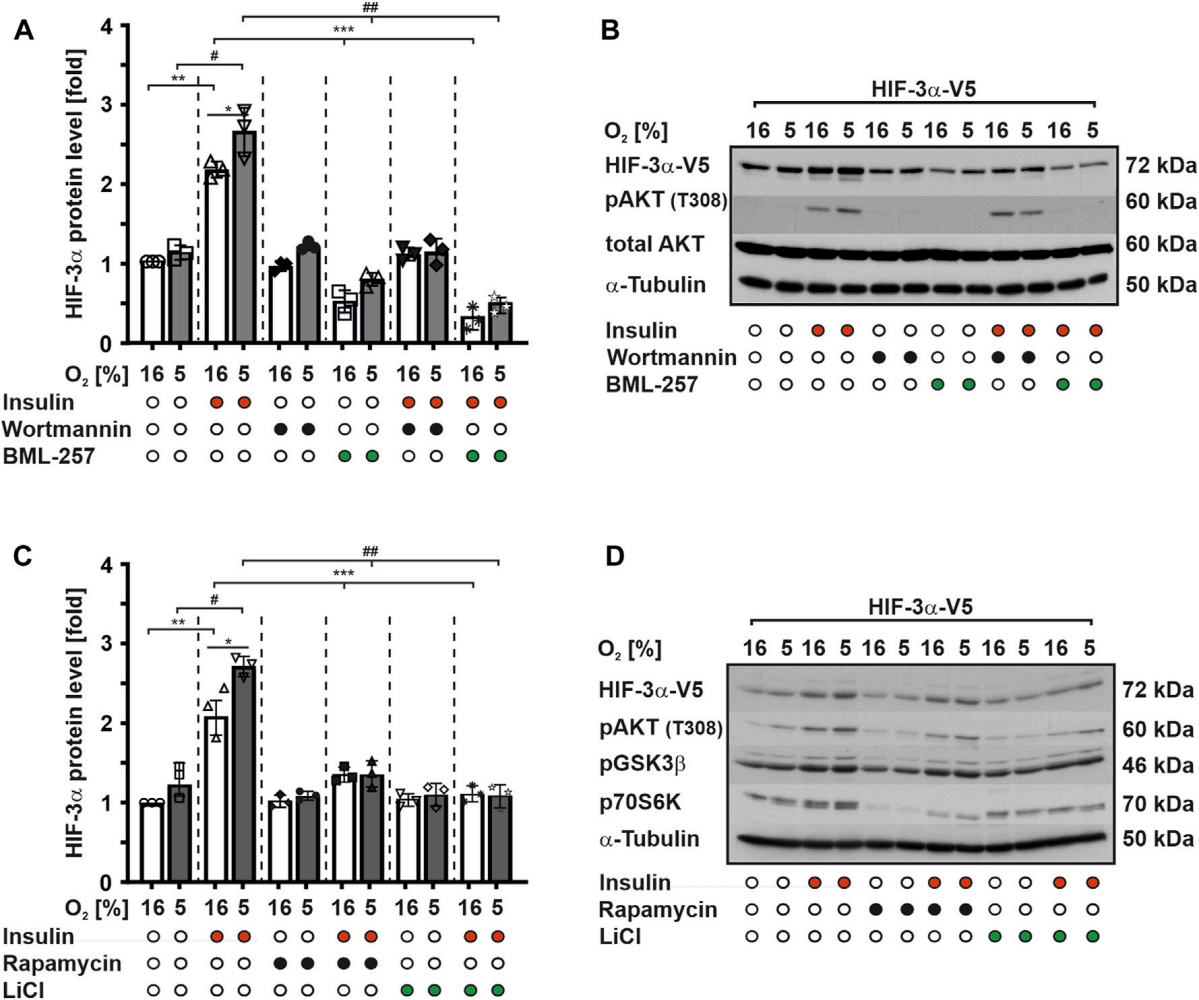
Insulin induces HIF-3α1 levels via PI3K/PKB signaling. MCF-7 cells were transfected with an expression vector for V5-tagged full-length HIF-3α1. After transfection, cells were starved in serum-free media for 16 h before being treated with insulin (100 nM), wortmannin (20 nM), BML-257 (12.5 µM), rapamycin (10 nM) or LiCl (500 nM) and further cultured under normoxia (16% O_2_) and mild hypoxia (5% O_2_) for 4 h. **(A,C)** HIF-3α1 protein levels were measured by Western blot analysis. HIF-3α1 protein levels in the control cells were set to 1. *significant differences 16% O_2_ vs 5% O_2_, **significant differences 16% O_2_ vs 16% O_2_ + insulin, ***significant differences 16% O_2_ + insulin vs 16% O_2_ + BML-257 or LiCl, ^#^significant differences between 5% O_2_ vs. 5% O_2_ + insulin, ^##^significant differences 5% O_2_ + insulin vs 5% O_2_ + BML-257 or LiCl, *p* ≤ 0.05, n = 3. **(B,D)** Representative Western blot analysis. 100 μg of total protein from MCF-7 cells were analyzed with antibodies against V5, pAKT (T308), total AKT, pGSK3β, p70S6K and α-Tubulin.

To investigate the potential role of the PI3K/PKB signaling pathway in insulin-mediated modulation of HIF-3α1 protein expression, we conducted experiments using MCF-7 cells because they express insulin receptors and respond to insulin stimulation (Kietzmann et al., 1999). First, we treated MCF-7 cells with insulin and demonstrated by qRT-PCR analysis that insulin upregulated *HIF3A* mRNA levels under both normoxia and mild hypoxia, which is in line with previous studies (Kietzmann et al., 2003; Heidbreder et al., 2007) (Supplementary Figure S1). Next, we transfected MCF-7 with wild-type V5-tagged HIF-3α1 and subsequently treated for a duration of 4 h with insulin, wortmannin (a potent PI3K inhibitor), and BML-257 (a specific PKB inhibitor) alone or in combination under normoxia or mild hypoxia. Our results demonstrated that treatment with insulin alone enhanced HIF-3α1 protein levels by about 2-fold under normoxia and by about 2.5-fold under mild hypoxia (Figures 1A, B). In contrast, when cells were treated with wortmannin alone or in combination with insulin no changes were observed in HIF-3α1 protein levels compared to the control cells, although PKB activation was inhibited (Figures 1A, B). This suggests that the insulin-induced HIF-3α1 expression involves the PI3K pathway.

However, treatment of cells with BML-257, a specific PKB inhibitor, alone resulted in a significant reduction of HIF-3α1 protein expression by about 50% under both normoxic and hypoxic conditions. Furthermore, the combined treatment of BML-257 and insulin did not reverse this decrease in HIF-3α1 protein levels (Figures 1A, B). Thus, these findings strongly suggest that the activation of HIF-3α1 protein expression by insulin is mediated through the PKB signaling pathway.

Next, we investigated whether PKB downstream targets, such as glycogen synthase kinase-3 (GSK-3) or mammalian target of rapamycin (mTOR), are involved in the regulation of HIF-3α1 protein expression. To achieve this, we treated MCF-7 cells with insulin and LiCl both well-known inhibitors of GSK-3 and rapamycin, a mTOR inhibitor alone or in combination under normoxia and mild hypoxia. Consistent with our previous findings, treatment with insulin alone increased HIF-3α1 protein expression under both normoxic and mild hypoxic conditions. However, treatment with LiCl and rapamycin alone slightly reduced HIF-3α1 protein levels compared to the control cells (Figures 1C, D). Furthermore, exposure of cells to these inhibitors in combination with insulin reduced insulin-mediated induction of HIF-3α1 protein levels but to a lesser extent than Wortmannin and BML-257 (Figures 1C, D). Collectively, these results suggest that PKB itself may regulate HIF-3α1 expression in response to insulin more potent and directly than the PKB downstream targets mTOR and GSK3.

### 3.2 HIF-3α1 is a direct target of PKB and serine 524 is crucial for its phosphorylation by PKB

**FIGURE 2:**
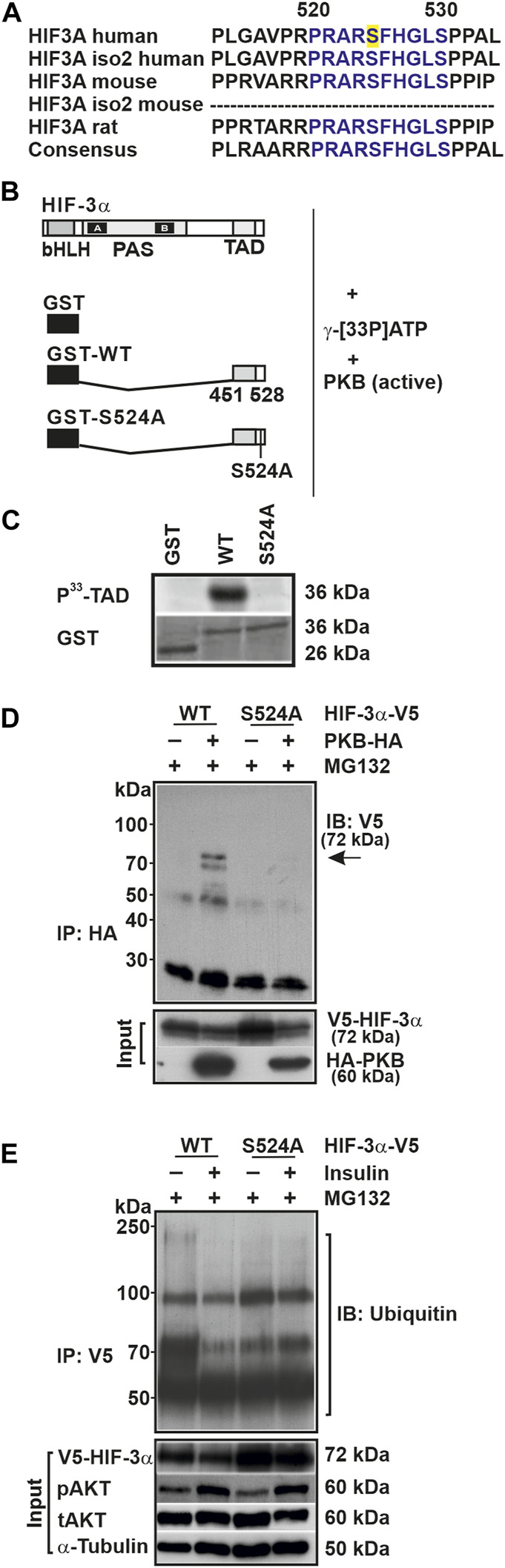
Serine 524 is essential for the phosphorylation of HIF-3α1 and its interaction with PKB. **(A)** Alignment of the amino acid sequences of HIF-3α isoforms from human, mouse, and rat. The conserved serine 524 residue is highlighted in yellow. **(B)** GST-HIF-3α-TAD wild-type (WT) or mutant (S524A) fusion proteins were prepared from *E. coli*, and 20 µg of these fusion proteins were incubated with 50 mU of active PKB/AKT1 and 10 µCi of [γ-^33^P]ATP for 30 min at 30°C. **(C)** Subsequently, the phosphorylated proteins were separated from unbound radioactivity by electrophoresis on a 10% SDS page gel. Radioactive proteins were visualized by phosphorimaging. **(D)** Immunoblot (IB) analysis of anti-HA (IP) and input from HEK 293 cells cotransfected with expression vectors for V5-tagged full-length HIF-3α1 WT or mutant (S524A) and HA-tagged PKB. Prior to cell lysis cells were treated with MG132 for 4 h. Blot from IP was analyzed with V5 antibody; inputs were probed with V5 or HA antibodies. **(E)** Immunoblot (IB) analysis of anti-V5 immunoprecipitates (IP) and input of HEK 293 cells treated with insulin and MG132 for 4 h after transfection with the indicated plasmids were analyzed with antibodies against ubiquitin, V5, pAKT, tAKT, and α-Tubulin.

To substantiate the finding that insulin is able to modulate HIF-3α1 protein expression via PKB, we first used computational approaches to search for the presence of putative PKB phosphorylation sites within HIF-3α1. We found that serine 524 matched the consensus motif, which is also highly conserved in mouse and rat HIF-3α (Figure 2A). This result was confirmed by mass spectrometry (MS) analysis of HIF-3 immunoprecipitates, which showed that serine 524 is indeed phosphorylated (Supplementary Figure S2). Notably, serine 524 is located within the transactivation domain (TAD), suggesting that phosphorylation of this residue may affect HIF-3α transactivity.

To investigate this, we transfected HepG2 cells with a Gal4 construct expressing a fusion protein with the wild-type TAD of HIF-3α (Gal4-HIF-3α-TAD) or a fusion protein in which the respective PKB site has been mutated (Gal4-HIF-3α-TAD-S/A). The cells were then treated with insulin for 8 h under normoxia and mild hypoxia. We found that mild hypoxia induced Luc activity by about 200% in both the wild-type and mutant construct (Supplementary Figure S3). Treatment with insulin enhanced Luc activity by about 600% under normoxia and by about 450% under mild hypoxia using the wild-type Gal4-HIF-3α-TAD construct, whereas insulin had no effect on the Luc activity on the mutant Gal4-HIF-3α-TAD-S/A construct (Supplementary Figure S3). Thus, insulin appears to regulate HIF-3α transactivity via serine 524.

To further support the idea that HIF-3α1 might be a direct target of PKB, we performed a phosphorylation assay. When using the GST-HIF-3α-TAD protein as a substrate we found that PKB phosphorylated the HIF-3α-TAD, whereas the GST protein alone was not phosphorylated (Figures 2B, C). Next, we used the GST-HIF-3α-TAD-S/A protein in which the putative PKB site was mutated and found that PKB no longer phosphorylated this protein (Figures 2B, C).

To validate the notion that HIF-3α1 is a direct target of PKB, we performed coimmunoprecipitation analysis to examine whether PKB forms a complex with HIF-3α1. Indeed, the results demonstrated an interaction between PKB and wild-type HIF-3α1 as they could be found in the same complex. Conversely, when lysates from cells expressing the HIF-3α1 S524A mutant were used, no interaction between PKB and HIF-3α1 could be detected (Figure 2D).

To determine whether the insulin-mediated HIF-3α1 regulation involves ubiquitination, we first performed coimmunoprecipitation analysis using proteins from HEK 293 cells transfected with vectors for V5-tagged wild-type HIF-3α1 or the HIF-3α1 S524A mutant. Subsequently, the cells were treated with insulin and the proteasome inhibitor MG132 for 4 h before harvesting. Although both wild-type and mutant HIF-3α1 proteins were found to be ubiquitinylated following immunoprecipitation with the V5-tag antibody, the extent of the ubiquitination was different. Treatment of cells with insulin inhibited the ubiquitination of wild-type HIF-3α1 whereas no differences in ubiquitination could be detected with the HIF-3α1 mutant upon insulin treatment (Figure 2E). In addition, we determined the half-life of HIF-3α1 and the respective PKB site mutant and could show that the HIF-3α1 S524A mutant´s half-life is prolonged compared to that of wild-type HIF-3α1 (Supplementary Figure S4). Together, these data suggest that insulin can modulate the protein expression of HIF-3α1 via PKB and that serine 524 is crucial for the binding of PKB within HIF-3α1 and may alter its protein stability.

### 3.3 HIF-3α1 affects cell colony-formation and cell adhesion

**FIGURE 3:**
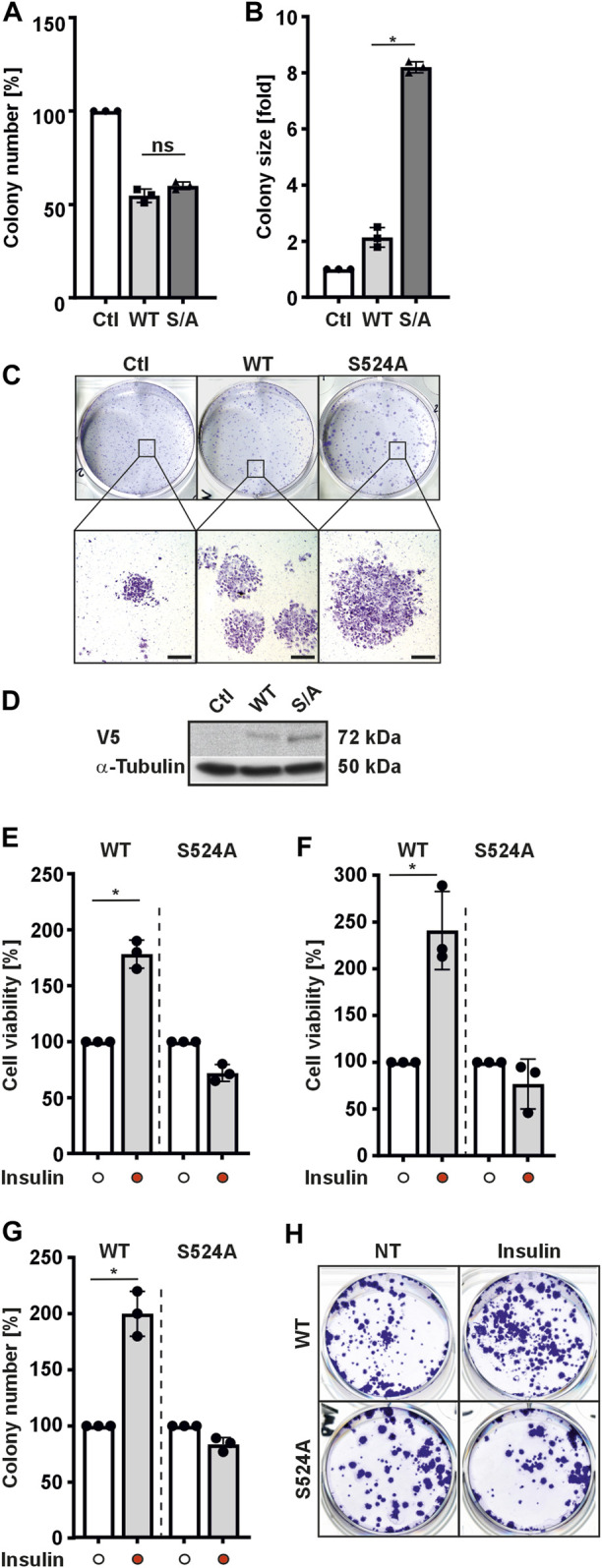
HIF-3α1 affects colony formation of MCF-7 and HEK 293 cells. MCF-7 cells were transfected with expression plasmids for V5-tagged wild-type HIF-3α1 (WT) or mutant (S524A). After transfection, cells were seeded onto 6-well plates at a density of 2000 cells/well and further cultured under normoxia (16% O_2_) for 7 days. Afterwards, cells were either fixed with 4% paraformaldehyde and stained with crystal violet or subjected to Western analysis. **(A)** Colony number and **(B)** colony size was analyzed. The numbers and sizes of colonies in the controls were set to 100% or 1, respectively. *significant differences HIF-3α1 WT vs HIF-3α1 S524A; *p* ≤ 0.05; n = 3. **(C)** Representative images of stained colonies. Scale bars: 500 µm **(D)** Representative Western Blots. 50 μg of total MCF-7 cell lysates were subjected to Western analysis with antibodies against V5 and α-Tubulin. **(E)** HEK 293 or **(F)** MCF-7 cells were transfected with expression plasmids for V5-tagged full-length HIF-3α1 WT or respective mutant (S524A). After transfection, cells were seeded onto 24-well plates at a density of 5 × 10^4^ cells/well, cultured under normoxia (16% O_2_) for 24 h and then treated with insulin in serum-free medium for 8 h. Thereafter, formation of MTT–formazane was measured at a wavelength of 570 nm using a Tecan plate reader. *significant differences HIF-3α1 WT vs HIF-3α1 WT + insulin; *p* ≤ 0.05; n = 3. **(G)** HEK 293 cells were transfected with expression plasmids for V5-tagged wild-type HIF-3α1 (WT) or mutant (S524A). After transfection cells were seeded onto 6-well plates at a density of 2000 cells/well and further cultured under normoxia (16% O_2_) for 5 days. Prior fixing, cells were cultured and treated with insulin (100 nM) in serum-free medium under normoxia (16% O_2_) for 48 h. Thereafter, cells were fixed with 4 % paraformaldehyde and stained with crystal violet and numbers of colonies were analyzed. The number of colonies in the untreated control (NT) were set to 100%.; *p* ≤ 0.05; n = 3. **(H)** Representative images of stained colonies.

HIF-3α has been proposed to play important roles in regulation of cell proliferation and growth (Tanaka et al., 2009; Ando et al., 2013; Xue et al., 2016; Zhou et al., 2018). The proper function of a protein in cells and tissues is tightly regulated by several mechanisms; of these, phosphorylation is one of the most important. Therefore, we next examined the effects of substituting serine 524 in HIF-3α1 on the ability of cells to form colonies. To this end, cells expressing either wild-type HIF-3α1 or the HIF-3α1 S524A mutant were seeded at low density and grown for 1 week before being subjected to crystal violet staining. Counting of cells showed that overexpression of HIF-3α1 variants (WT and S/A) reduced the number of colonies compared to the control (Ctl) (Figure 3A). Interestingly, overexpression of HIF-3α1 WT produced about 2.5-fold larger colonies compared to the control (Ctl). Notably, transfection of cells with the HIF-3α1 S524A induced even more widespread colony growth, where the colony size was about 8-fold larger in comparison to that of the control cells (Figures 3B–D). In line with this, the reduction in the number of colonies as well as the production of larger colonies could be completely abolished by using two different siRNAs against HIF-3α1 (Supplementary Figure S5).

Next, we investigated whether insulin might have an influence on the HIF-3α1 modulated cell viability as well as colony formation. Again, HIF-3α1 WT and the corresponding HIF-3α1 S524A mutant expressing cells were used. We found that application of insulin improved the cell viability of HIF-3α1 WT transfected HEK 293 cells by approximately 180% and increased it by approximately 240% in MCF-7 transfected cells. (Figures 3E, F). In addition, the number of colonies in HIF-3α1 WT transfected cells increased upon insulin treatment (Figures 3G, H). In contrast, treatment of cells with insulin did not affect viability or colony number of cells transfected with the HIF-3α1 S524A mutant (Figures 3E–H). Thus, these findings further support the conclusion that serine 524 is crucial for the insulin-mediated regulation of HIF-3α1.

**FIGURE 4:**
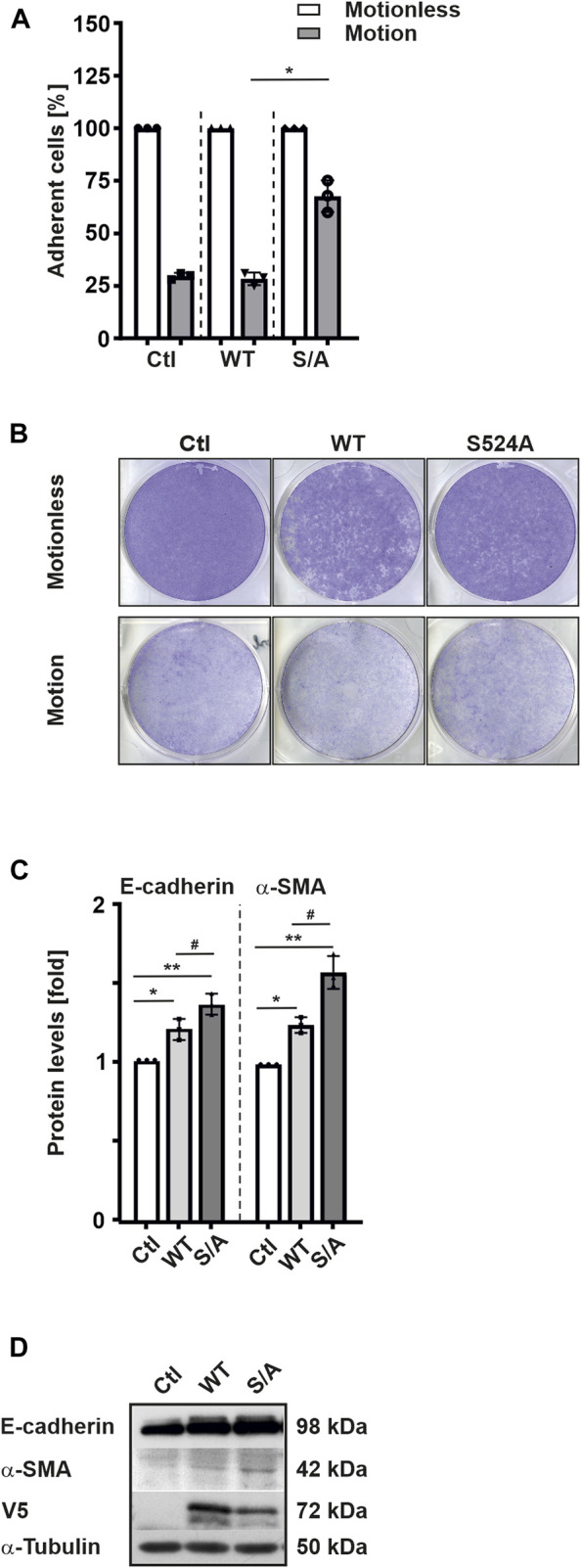
HIF-3α1 affects cell adhesion in MCF-7 cells. MCF-7 cells were transfected with expression plasmids for full-length V5-tagged HIF-3α1 (WT) or mutant (S524A), seeded onto 6 well-plates and grown under normoxia (16% O_2_) until they reached >90% confluency. The plates were then rotated at 250 rpm on an orbital shaker for 8 h at 37°C. Then, cells were fixed with 4% paraformaldehyde and stained with crystal violet. **(A)** The strength of adhesion is represented as a percentage of the number of cells remaining after rotation. The relative cell number without motion was set to 100%. *significant differences HIF-3α1 WT (motion) vs HIF-3α1 S524A (motion); *p* ≤ 0.05; n = 3. **(B)** Representative images of cells remaining attached to the wells. **(C)** Protein levels were measured by Western blot analysis. *significant differences control (Ctl) vs HIF-3α1 WT, **significant differences Ctl vs HIF-3α1 S524A, ^#^significant differences HIF-3α1 WT vs HIF-3α1 S524A. The protein level in the respective control was set to 1; *p* ≤ 0.05; n = 3. **(D)** Representative Western Blots. 50 μg of total cell lysates were subjected to Western analysis with antibodies against V5, E-cadherin, α-SMA, and α-Tubulin.

Since we observed that overexpression of HIF-3α1 WT or the HIF-3α1 S524A mutant reduced colony number but increased colony size compared to control cells, we next investigated whether HIF-3α1 also affects cell adhesion. To do this, we transfected cells with HIF-3α1 WT or the HIF-3α1 S524A mutant and subjected them to rotation on an orbital shaker for 8 h. After rotation, ∼70% of the control cells were detached. Similarly, cells overexpressing HIF-3α1 WT showed a comparable level of adhesion (Figures 4A, B). By contrast, only about 40% of cells were detached when the HIF-3α1 S524A mutant construct was used (Figures 4A, B). Additionally, we investigated the expression of E-cadherin which plays an important role in cell-cell adhesion and tissue formation as well as of α-smooth muscle actin (α-SMA), a protein constituent of the contractile apparatus. Overexpression of full-length HIF-3α1 WT appeared to induce both E-cadherin and α-SMA expression (Figures 4C, D). Consistent with the reduced detachment in adhesion assay, the highest expression levels of these two proteins were seen in cells expressing the HIF-3α1 S524A mutant (Figures 4C, D).

### 3.4 Insulin promotes HIF-3α1-induced migration

**FIGURE 5:**
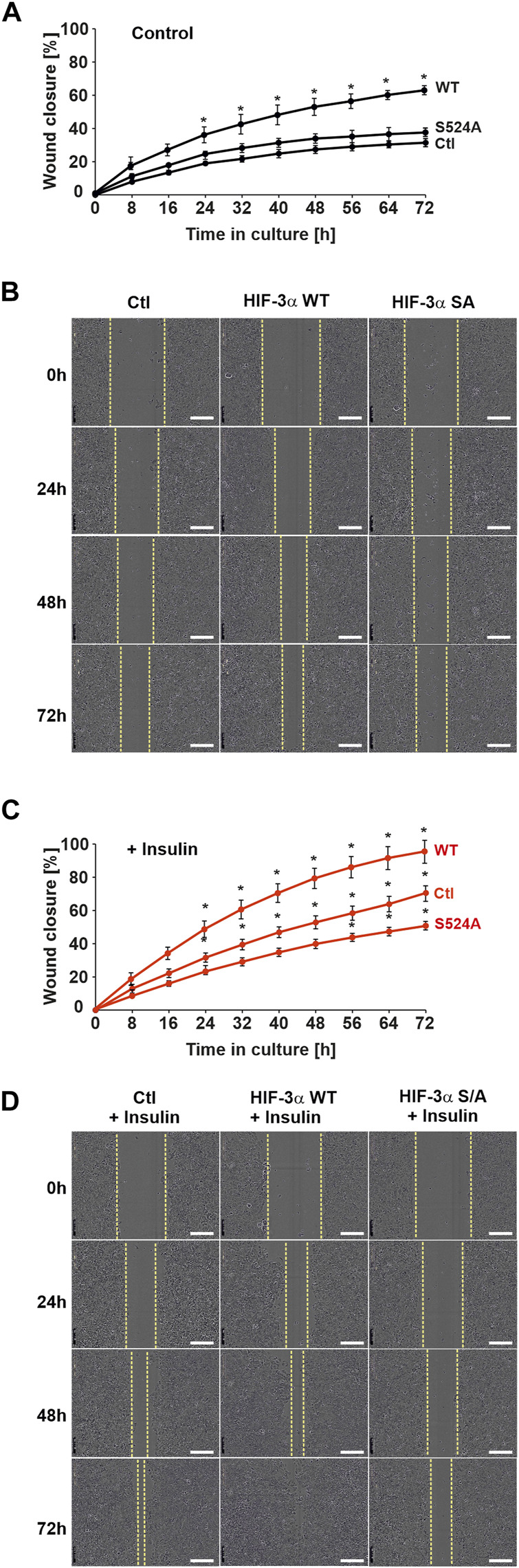
Insulin promotes HIF-3α1-induced migration in a wound healing assay. MCF-7 cells were transfected with expression plasmids for V5-tagged full-length HIF-3α1 WT or HIF-3α1 (S524A) mutant. 24 h post transfection, cells were seeded onto 96 well-plates and grown until they reached confluency. Wounding was induced by scratching the cell monolayer with a wound maker and cells were then further cultured in medium supplemented with 0.1% FBS + insulin. **(A,C)** Real-time wound closure of HIF-3α1 WT and HIF-3α1 S524A mutant with or without insulin treatment, *significant differences control (Ctl) vs HIF-3α1 WT or HIF-3α1 S524A, *p* ≤ 0.05, n = 3. **(B,D)** Representative images of wound closure. Scale bars: 300 µm.

The above findings indicated that HIF-3α1 and its insulin-dependent regulation may play a role in cell adhesion as well as colony-forming ability of cells. Next, it was investigated whether HIF-3α1 mediates the mobility of cells in a wound healing assay. The results of the wound healing assay showed that cells overexpressing wild-type HIF-3α1, but not the HIF-3α1 S524A mutant, migrated 2-fold faster than non-transfected cells (Figures 5A, B). As expected, exposure of wild-type HIF-3α1 transfected cells with insulin promoted cell migration and increased the rate of wound closure by about 40% and closed the wound in 72–74 h when compared with the control (Figures 5C, D), whereas insulin was able to cause a decrease in the rate of wound closure in HIF-3α1 S524A transfected cells in comparison to the control (Figures 5C, D). Overall, these findings offer the potential to affect HIF-3α-dependent processes by targeting PKB.

## 4 Discussion

The present study aimed to investigate the role of insulin in the regulation of HIF-3α1 and its potential impact on cell adhesion, colony formation, cell viability, and migration. The data revealed involvement of the PI3K/PKB signaling pathway in insulin-dependent HIF-3α1 expression where serine 524 was identified as a crucial phosphorylation site for PKB. The mutation of serine 524 was found to affect the regulation and activity of HIF-3α1, suggesting its importance in cancer-related processes, although evidence in mice *in-vivo* is still pending.

The HIF family plays a crucial role in cellular adaptations to low oxygen levels and is involved in various cellular processes, including erythropoiesis, glucose metabolism, angiogenesis, and cancer [for review, see (Semenza, 2016; Kietzmann, 2020; Kierans and Taylor, 2021; Wicks and Semenza, 2022)]. Although HIF-1α and HIF-2α have been extensively studied, the function of HIF-3α isoforms remains poorly characterized. Initial findings suggested that hypoxia can induce the expression of some HIF-3α variants via HIF-1α but not HIF-2α in a cell and tissue-specific manner (Li et al., 2006; Heidbreder et al., 2007; Tanaka et al., 2009; Yang et al., 2015) and that these HIF-3α isoforms act as negative regulators of the hypoxia response by competing with HIF-1α for binding to HIF-1β/ARNT (Hara et al., 2001; Heikkilä et al., 2011). However, recent studies have shown that full-length HIF-3α1 and the long splicing variant HIF-3α2 can act as positive regulators and induce the expression of some genes. These include erythropoietin (EPO) (Tolonen et al., 2020), and the DNA damage inducible transcript 3 (DDIT4) gene (Jaśkiewicz et al., 2022). Furthermore, HIF-3α1 transgenic mice showed upregulation of genes whose products are critically involved in lung development (Huang et al., 2013). In zebrafish, *Hif-3α* was shown to upregulate hypoxia-inducible expression of a large number of genes, including igfbp-1b, mclb, sqrd, and zp3v2 (Zhang et al., 2014; Duan, 2016). Similar transcriptional activity was observed for HIF-3α1 and HIF-3α9 in primary rat hepatocytes and other cell types, respectively (Scharf et al., 2005; Zhang et al., 2014).

Previous evidence indicated that, similar to HIF-1/2 α-subunits, long HIF-3α variants contain a conserved ODD domain and are therefore potential targets for PHD- and VHL-dependent degradation (Maynard et al., 2003; Heikkilä et al., 2011). However, the current study could not support this as hypoxia treatment did not significantly affect HIF-3α1 protein expression. This aligns with other studies demonstrating that HIF-3α levels remain unaffected by hypoxia in COS-7 and HeLa cells (Hara et al., 2001; Augstein et al., 2011). Additionally, long HIF-3α isoforms were found in both the nucleus and cytoplasm under normal oxygen conditions in various models (Heikkilä et al., 2011; Ravenna et al., 2016). On the other hand, *in-vitro* ubiquitination assays showed that addition of cellular extracts devoid of the 26S proteasome containing wild-type pVHL efficiently polyubiquitinated the HIF-3α1 [ODDD] (encoded by the full-length ODD domain only), whereas full-length HIF-3α1 was less robustly ubiquitinated (Maynard et al., 2003). These studies suggested that other structural elements besides the ODDD might be required for the hypoxia-dependent modulation of full-length HIF-3α1 protein stability. Moreover, studies investigating the hypoxic induction of HIF-3α1 have yielded inconsistent results. While experiments with lung epithelial cells suggested that both mRNA and protein levels of endogenous HIF-3α were induced by acute hypoxia, other studies using different cell types and oxygen concentrations did not observe a similar response which may be due to the isoform- and cell type-specific regulation as well as the different oxygen concentrations used (Li et al., 2006; Augstein et al., 2011).

Interestingly, non-hypoxic stimuli such as insulin-like growth factors (Zelzer et al., 1998; Feldser et al., 1999; Stiehl et al., 2002), insulin (Treins et al., 2002; Kietzmann et al., 2003) interleukin-1, tumor necrosis factor-α (Hellwig-Bürgel et al., 1999; Thornton et al., 2000), angiotensin II (Richard et al., 2000), and thrombin (Görlach et al., 2001) are capable of regulating HIFα isoforms. While it was previously demonstrated that insulin can stimulate endogenous HIF-3α in rat hepatocytes through the PI3K/AKT signaling pathway under both normoxic and mild hypoxic conditions (Kietzmann et al., 2003), there was so far no supporting evidence indicating that the HIF-3α subunits can be directly phosphorylated by PKB/AKT. However, our current findings indicate that HIF-3α1 induction by insulin is completely abolished by a specific PKB inhibitor and mutation at S524. This suggests that PKB directly acts on HIF-3α1 during insulin activation.

Typically, PKB phosphorylates proteins containing the consensus motif R-X-R-XX-S/T-F/L, where X represents any amino acid residue (Alessi and Cohen, 1998). By using a computer-based approach, we identified amino acid serine 524 located in the ODDD of HIF-3α1 as a potential PKB phosphorylation site. Indeed, conversion of S524 to alanine completely abolished HIF-3α1 phosphorylation as well as binding of PKB. Further, ubiquitination experiments with the proteasomal inhibitor MG132 revealed that phosphorylation of serine 524 by PKB results in a reduced ubiquitination of HIF-3α1 upon insulin treatment concomitant with the finding that insulin enhanced HIF-3α1 levels. Consistently, the S524A mutation significantly increased HIF-3α1 protein stability. Of note, this occurred even in the absence of insulin, suggesting that PKB phosphorylation may also regulate HIF-3α1 expression independent of insulin. As activation of PI3K/PKB signaling can occur by growth factors other than insulin, this suggests that serine 524 may be targeted by PKB in response to a variety of PI3K/PKB acting hormones and growth factors, which need to be further elucidated.

Our study also uncovered a previously unknown function of HIF-3α1 in colony formation, cell viability, and migration which are important in tumorigenesis. Previous studies have primarily focused on HIF-1/2α, and little is known about the role of HIF-3α in those processes (Pouysségur et al., 2006; Rankin and Giaccia, 2008; Lv et al., 2016). However, upregulation of long HIF-3α variants has been frequently observed in various cancer cell lines and may induce the expression of genes involved in tumor progression or aggressiveness [for review, see (Lv et al., 2016)]. Full-length HIF-3α variants, such as HIF-3α1 and HIF-3α9, have transactivation activity and can activate a large number of target genes in response to hypoxia whereas the short isoform HIF-3α4 has been shown to act as a dominant-negative regulator of HIF-1/2α (Makino et al., 2001; Maynard et al., 2005; Heikkilä et al., 2011) that is able to impair proliferation and angiogenesis in hypervascular meningioma cells (Ando et al., 2013).

Notably, HIF-3α1, but not HIF-1α, has been shown to upregulate genes in the Janus kinase-signal transducer and activator of transcription (JAK-STAT) signaling pathway, which is critical in promoting carcinogenesis in colorectal cancer tissues (Zhang et al., 2014). Recent studies also suggest that HIF-3α1 activates JAK-STAT3 signaling via a transcription-independent mechanism, promoting colorectal tumor cell growth (Xue et al., 2016). Additionally, knockout studies in pulmonary endothelial cells have indicated that HIF-3α disruption impairs proliferative and angiogenic activities, which can be restored by expressing full-length HIF-3α cDNA, regardless of oxygen tension (Kobayashi et al., 2015). Consistent with these findings, our study demonstrated that overexpression of HIF-3α1 enhances colony formation under normoxia. Considering that the HIF-3α1 S524A mutant, which has increased stability compared to the wild-type, did not disrupt the promoting effect on cell growth, it appears that PKB phosphorylation of HIF-3α1 not only alters protein stability but also modulates its activity.

Changes in cell adhesion and migration are associated with alterations in the expression of several key proteins involved in cell-cell interactions (e.g., E-cadherin) and the cytoskeleton (e.g., α-SMA). While overexpression of HIF-3α1 WT did not affect cell adhesion significantly, it promoted cell migration in a wound healing assay. This is consistent with a recent study showing that HIF-3α promotes pancreatic cancer cell invasion and migration under both normoxia and hypoxia (Zhou et al., 2018). Interestingly, the HIF-3α1 S524A mutant induced the expression of E-cadherin and α-SMA while it even slightly inhibited cell migration. This might be due to the paradoxical roles of E-cadherin in regulating tumorigenicity, which is observed in many types of cancer, suggesting that maintaining E-cadherin expression and cell-cell adhesion is crucial for cancer cell invasion and metastasis (Chu et al., 2013).

In conclusion, our study provides evidence that HIF-3α1 is regulated by PKB/AKT-mediated phosphorylation, which affects its stability and activity. Furthermore, HIF-3α1 influences colony formation, cell viability, and migration, suggesting its potential role in tumorigenesis. Future investigations should focus on elucidating the HIF-3α1 downstream pathways and their precise contribution to cellular functions.

## Data Availability

The original contributions presented in the study are included in the article/Supplementary Material, further inquiries can be directed to the corresponding authors.

## Supplementary material

SUPPLEMENTARY FIGURE S1 Insulin induced *HIF3A* mRNA levels. MCF-7 cells were cultured under normoxic (16% O_2_) conditions for 24 h, then treated with insulin (100 nM) and further cultured in serum-free media either under normoxia or mild hypoxia (5% O_2_) for 8 h. Relative *HIF3A* mRNA levels were evaluated by quantitative real-time PCR relative to β-actin mRNA in triplicate samples. *significant differences 16% O_2_ vs. 16% O_2_ + insulin, **significant differences 5% O_2_ vs 5% O_2_ + insulin, *p* ≤ 0.05, n = 3.

SUPPLEMENTARY FIGURE S2 Lumos Fusion Orbitrap mass spectra of trypsin digests of expressed HIF-3α1.

SUPPLEMENTARY FIGURE S3 Insulin modulates HIF-3α transactivity. HepG2 cells were cotransfected with vectors for Gal4-HIF-3α-TAD or Gal4-HIF-3α-TAD-S/A mutant and the p5GE1B-Luc gene construct. After transfection, cells were cultured with fresh medium for 48 h under normoxia (16% O_2_). Thereafter, cells were treated with insulin and cultured in serum-free medium for the next 8 h under normoxic (16% O_2_) and mild hypoxic (5% O_2_) conditions. In each experiment, the percentage of Luc activity relative to the control was set to 100%. The values represent means + SEM of results from three independent experiments. *significant differences 16% O_2_ vs 5% O_2_, **significant differences 16% O_2_ vs. 16% O_2_ + insulin, ^#^significant differences 5% O_2_ vs. 5% O_2_ + insulin, *p* ≤ 0.05, n = 3.

SUPPLEMENTARY FIGURE S4 Mutation of serine 524 prolongs the half-life of HIF-3α1. **(A)** HEK 293 cells were transfected with expression vectors for V5-tagged full-length HIF-3α1 wild-type (WT) or HIF-3α1 mutant (S524A) and cultured under normoxia (16% O_2_). After inhibition of protein synthesis with cycloheximide (CHX; 20 mg/mL), cells were lysed at indicated time points and HIF-3α1 protein half-life was measured by Western blot analysis. The HIF-3α1 protein levels at 0 h were set to 100%. *significant differences WT vs S524A, *p* ≤ 0.05, n = 3. **(B)** Representative Western blot analysis. 100 µg of total protein lysates from HEK 293 cells were analyzed with antibodies against V5 and α-Tubulin.

SUPPLEMENTARY FIGURE S5 Knockdown of HIF-3α1 affects colony formation in MCF-7 cells. MCF-7 cells were transfected with two different siRNAs against HIF-3α1. 48 h post-transfection, cells were seeded on 6-well plates at a density of 2,000 cells/well and cultured for 7 days under normoxia (16% O_2_). Afterwards, cells were fixed with 4% paraformaldehyde and stained with crystal violet. **(A)** Colony number and **(B)** colony size were analyzed. The numbers and sizes of colonies in the controls were set to 100%. *significant differences Ctl vs siHIF-3α_1, **significant differences Ctl vs siHIF-3α_2; , *p* ≤ 0.05, n = 3. **(C)** Representative images of stained colonies. Scale bars: 500 µm.
